# Future perspectives in melanoma research: meeting report from the “Melanoma Bridge”: Napoli, December 3rd–6th 2014

**DOI:** 10.1186/s12967-015-0736-1

**Published:** 2015-11-30

**Authors:** Paolo A. Ascierto, Michael Atkins, Carlo Bifulco, Gerardo Botti, Alistair Cochran, Michael Davies, Sandra Demaria, Reinhard Dummer, Soldano Ferrone, Silvia Formenti, Thomas F. Gajewski, Claus Garbe, Samir Khleif, Rolf Kiessling, Roger Lo, Paul Lorigan, Grant Mc Arthur, Giuseppe Masucci, Ignacio Melero, Martin Mihm, Giuseppe Palmieri, Giorgio Parmiani, Igor Puzanov, Pedro Romero, Bastian Schilling, Barbara Seliger, David Stroncek, Janis Taube, Sara Tomei, Hassane M. Zarour, Alessandro Testori, Ena Wang, Jérôme Galon, Gennaro Ciliberto, Nicola Mozzillo, Francesco M. Marincola, Magdalena Thurin

**Affiliations:** Istituto Nazionale Tumori, Fondazione “G. Pascale”, Naples, Italy; Georgetown-Lombardi Comprehensive Cancer Center, Washington, DC, USA; Translational Molecular Pathology, Earle A. Chiles Research Institute, Providence Cancer Center, Portland, OR USA; Departments of Pathology and Laboratory Medicine and Surgery, David Geffen School of Medicine at University of California Los Angeles (UCLA), John Wayne Cancer Institute, Santa Monica, CA USA; Department of Melanoma Medical Oncology, Division of Cancer Medicine, The University of Texas MD Anderson Cancer Center, Houston, TX USA; Departments of Radiation Oncology and Pathology, Weill Cornell Medical College, New York, NY USA; Skin Cancer Unit, Department of Dermatology, University Hospital Zürich, 8091 Zurich, Switzerland; Department of Surgery, Massachusetts General Hospital, Harvard Medical School, Boston, MA USA; Department of Radiation Oncology, Weill Cornell Medical College, New York, NY USA; Departments of Medicine and of Pathology, Immunology and Cancer Program, The University of Chicago Medicine, Chicago, IL USA; Department of Dermatology, Center for Dermato Oncology, University of Tübingen, Tübingen, Germany; Georgia Regents University Cancer Center, Georgia Regents University, Augusta, GA USA; Department of Oncology-Pathology, Karolinska Institute, Stockholm, Sweden; Departments of Medicine and Molecular and Medical Pharmacology, David Geffen School of Medicine and Jonsson Comprehensive Cancer Center at the University of California Los Angeles (UCLA), Los Angeles, CA USA; University of Manchester/Christie NHS Foundation Trust, Manchester, UK; Peter MacCallum Cancer Centre and University of Melbourne, Victoria, Australia; Department of Oncology-Pathology, The Karolinska Hospital, Stockholm, Sweden; Centro de Investigación Médica Aplicada, and Clinica Universidad de Navarra, Pamplona, Navarra Spain; Department of Dermatology, Harvard Medical School, Boston, MA USA; Unit of Cancer Genetics, Institute of Biomolecular Chemistry, National Research Council, Sassari, Italy; Division of Molecular Oncology, Unit of Bio-Immunotherapy of Solid Tumors, San Raffaele Institute, Milan, Italy; Vanderbilt University Medical Center, Nashville, TN USA; Ludwig Cancer Research Center, University of Lausanne, Lausanne, Switzerland; Department of Dermatology, University Hospital, West German Cancer Center, University Duisburg-Essen, Essen, Germany; Institute of Medical Immunology, Martin Luther University Halle-Wittenberg, Halle, Germany; Cell Processing Section, Department of Transfusion Medicine, Clinical Center, NIH, Bethesda, MD USA; Department of Dermatology, Johns Hopkins University SOM, Baltimore, MD USA; Division of Translational Medicine, Sidra Medical and Research Center, Doha, Qatar; Departments of Medicine, Immunology and Dermatology, University of Pittsburgh, Pittsburgh, PA USA; Istituto Europeo di Oncologia, Milan, Italy; Division of Translational Medicine, Sidra Medical and Research Centre, Doha, Qatar; INSERM, UMRS1138, Laboratory of Integrative Cancer Immunology, Université Paris Descartes, Sorbonne Paris Cité, Centre de Recherche des Cordeliers, Paris, France; Sidra Medical and Research Centre, Doha, Qatar; Cancer Diagnosis Program, National Cancer Institute, NIH, Bethesda, MD USA; German Cancer Consortium (DKTK), Essen, Germany

## Abstract

The fourth “Melanoma Bridge Meeting” took place in Naples, December 3–6th, 2014. The four topics discussed at this meeting were: Molecular and Immunological Advances, Combination Therapies, News in Immunotherapy, and Tumor Microenvironment and Biomarkers. Until recently systemic therapy for metastatic melanoma patients was ineffective, but recent advances in tumor biology and immunology have led to the development of new targeted and immunotherapeutic agents that prolong progression-free survival (PFS) and overall survival (OS). New therapies, such as mitogen-activated protein kinase (MAPK) pathway inhibitors as well as other signaling pathway inhibitors, are being tested in patients with metastatic melanoma either as monotherapy or in combination, and all have yielded promising results. These include inhibitors of receptor tyrosine kinases (BRAF, MEK, and VEGFR), the phosphatidylinositol 3 kinase (PI3K) pathway [PI3K, AKT, mammalian target of rapamycin (mTOR)], activators of apoptotic pathway, and the cell cycle inhibitors (CDK4/6). Various locoregional interventions including radiotherapy and surgery are still valid approaches in treatment of advanced melanoma that can be integrated with novel therapies. Intrinsic, adaptive and acquired resistance occur with targeted therapy such as BRAF inhibitors, where most responses are short-lived. Given that the reactivation of the MAPK pathway through several distinct mechanisms is responsible for the majority of acquired resistance, it is logical to combine BRAF inhibitors with inhibitors of targets downstream in the MAPK pathway. For example, combination of BRAF/MEK inhibitors (e.g., dabrafenib/trametinib) have been demonstrated to improve survival compared to monotherapy. Application of novel technologies such sequencing have proven useful as a tool for identification of MAPK pathway-alternative resistance mechanism and designing other combinatorial therapies such as those between BRAF and AKT inhibitors. Improved survival rates have also been observed with immune-targeted therapy for patients with metastatic melanoma. Immune-modulating antibodies came to the forefront with anti-CTLA-4, programmed cell death-1 (PD-1) and PD-1 ligand 1 (PD-L1) pathway blocking antibodies that result in durable responses in a subset of melanoma patients. Agents targeting other immune inhibitory (e.g., Tim-3) or immune stimulating (e.g., CD137) receptors and other approaches such as adoptive cell transfer demonstrate clinical benefit in patients with melanoma as well. These agents are being studied in combination with targeted therapies in attempt to produce longer-term responses than those more typically seen with targeted therapy. Other combinations with cytotoxic chemotherapy and inhibitors of angiogenesis are changing the evolving landscape of therapeutic options and are being evaluated to prevent or delay resistance and to further improve survival rates for this patient population. This meeting’s specific focus was on advances in combination of targeted therapy and immunotherapy. Both combination targeted therapy approaches and different immunotherapies were discussed. Similarly to the previous meetings, the importance of biomarkers for clinical application as markers for diagnosis, prognosis and prediction of treatment response was an integral part of the meeting. The overall emphasis on biomarkers supports novel concepts toward integrating biomarkers into contemporary clinical management of patients with melanoma across the entire spectrum of disease stage. Translation of the knowledge gained from the biology of tumor microenvironment across different tumors represents a bridge to impact on prognosis and response to therapy in melanoma.

## Background

Opening ceremony included the lecture of Giorgio Parmiani, MD who was the 2014 awardee from the FONDAZIONE MELANOMA ONLUS that is one of the sponsors of the meeting. Awards are presented each year for outstanding scientific achievements in the field of melanoma.

The Fondazione awarded Giorgio Parmiani in recognition of his outstanding research and achievements in cancer therapy. Giorgio Parmiani holds an MD degree from the University of Milan and he is currently the Head of the Unit of Immuno-Biotherapy of Melanoma and Solid Tumors at the San Raffaele Foundation Scientific Institute. Dr. Parmiani’s research interests have been focused on the study of molecular characterization of human tumor Antigens and the T cell response, particularly in melanoma patients. His interests have also focused on studies of immunotherapy in melanoma, colorectal, and prostate cancer patients, primarily with gene-modified cellular vaccines, along with peptides or heat-shock protein-based vaccines. Dr. Parmiani presented the Award lecture during the Opening Ceremony.

### Giorgio Parmiani award lecture

Anti-tumor immune response have to be considered as a dynamic system, where the activation of B and T lymphocytes (helper CD4+ and cytotoxic CD8+, respectively) is counterbalanced by the suppression from T-regulatory (Treg), dysfunctional myeloid cells (myeloid-derived suppressor cells, MDSC); immature dendritic cells (iDC) and T and B “exhausted” or anergic lymphocytes. In primary melanoma, the density and distribution of tumor-infiltrating lymphocytes (TILs) is a positive and independent prognostic factor. Patients with grade 3 TILs infiltration, defined as dense and diffuse lymphocytes infiltrate throughout the tumor have better survival as compared with those without infiltration [1]. These findings indicated the potential of the therapeutic use of vaccines in metastatic melanoma (MM) patients. Both normal subjects and melanoma patients show tolerance to “self” melanoma-associated antigens (MAAs). Tolerance needs to be broken in order to induce a T-cell immune response against “self” MAAs which are considered to be “weak” antigens.

Most common MAAs recognized by T-cells including melanoma differentiation antigens (e.g. MART-1, GP100), cancer testis antigens (e.g., MAGE-1, NY-ESO-1), mutated antigens (neo-antigens) and oncogenic transformation-associated antigens including oncogenes (BRAF, survivin, telomerase) are overexpressed in melanoma. Early stages of melanoma vaccines development (1995–2008) have been based on whole cell and peptide-specific antigens such as MAGE-3 and Melan-A/MART-1. Clinical trials results of first generation self-peptide-based vaccination in MM patients were dismal (1998–2008). Phase I–II trials with vaccines in MM patients showed less than 20 % of objective response rate (ORR) but 20–65 % of immune response rate (IRR) [2]. Dendritic cells (DC) presented antigens have been demonstrated to be superior to peptides as MAGE-3.A1-based vaccine. Nonetheless, only few patients experienced an immune response as well as partial or complete clinical responses to vaccine therapy [3]. However, positive overall response (OS) in a phase 3 study have been reached with GP100 [4] and dendritic cells-based vaccines [5] although such responses were delayed and were often long lasting.

In the following years, due to the scientific advances in immunology and tumor biology, clinical trials generated more promising results for treatment of melanoma patients with vaccines [4–7]. New generation vaccines, developed using different strategies, showed more promising results in patients with solid tumors, including MM. A systematic review of new generation vaccines, including 4375 patients from 56 clinical trials in MM, showed an overall disease control rate (DCR) in 25 % of patients [8]. The presence of a tumor-specific immune response was associated with prolonged overall survival but there was no evidence that anti-melanoma vaccines provide better OS as compared to other treatments.

The lack of predictive factors for efficacy that will allow to select patients for treatment is a critical factor and a challenge for melanoma immunotherapy. In an adjuvant phase III trial (DERMA trial) which enrolled 1349 radically resected, stage IIIB–IIIC melanoma patients, MAGE-A3 vaccine did not reach its primary endpoint of disease-free survival (DFS) prolongation as compared with observation but a gene signature was developed with a potential to predict a response to the vaccine [9].

Other important options in the development of melanoma vaccines are the combination with chemotherapy and with molecularly targeted treatment agents (e.g., vemurafenib) that can also modulate the immune system. New immunotherapeutic approaches such as immuno-checkpoint inhibitors (anti-CTLA4, anti-PD-1, and anti-PD-L1) that already have changed the strategies of MM treatment due to their therapeutic efficacy should also be considered. Accumulation of immunosuppressive cells in the blood [such as MDSC cells and Treg is associated with advanced disease stage in melanoma and in prostate cancer patients [10]. One of the most effective immune-stimulatory mechanisms would be down-regulation of both Treg cells and MDSC, which can result from vaccination against MAAs. Preliminary results showed that a treatment with ipilimumab could help triggering a clinically effective MAA-specific response on patients with MM targeting self/shared and/or mutated antigens.

Given the recent progress regarding genome sequencing technologies, the possibility of identifying new somatic mutation in genes encoding tumor-specific antigens for individual patient allow to build personalized melanoma vaccines. Immune responses to peptide sequences derived from neo-antigens was observed in melanoma patients who demonstrated a response to CTLA-4 blockade [11]. These findings provide a rationale for exome sequencing to identify mutational load as predcictive marker of response for patients for whom checkpoint blockade therapy is considered,

## Combination therapies

### Targeted therapy combinations

Melanoma is a genetically heterogeneous disease, with oncogenic driver mutations which are present in most tumors. The Cancer Genome Atlas Research (TCGA) Network established four subtypes of cutaneous melanoma based on mutation in B*RAF*, *RAS*, *NF1* genes and Triple Wild-Type. Tumors driven by V600-BRAF mutations that are highly sensitive to RAF-inhibitors. *RAS* and *NF1* mutant melanomas have deregulated MEK signaling may be responsive to MEK inhibitors. *RAS*, *NF1*, and Triple Wild-Type cancers all demonstrated overexpression of *AKT3*, a protein kinase that affects MEK and mTOR signaling pathways, suggesting that MEK and PI3K/AKT/mTOR pathway inhibitors could target this molecular alteration. Patients with metastatic melanoma with greater numbers of immune cells infiltrating tumors in the lymph nodes and enhanced T-cell signaling experienced better outcomes.

The plasticity of human tumor cells generally replicates normal molecular processes occurring during development and tissue repair. In humans, cancer progression is also shaped by host immune responses that edit the final tumor-host interactions. The genetic complexity and extreme variability of human melanoma means a multidisciplinary integrative approach is needed to understand the interactions between the genetic background of the host, the tumor and its microenvironment, and the impact of these on the immune system [12]. It is evident that successful anti-tumor strategies need to encompass a multimodal approach to avoid tumor escape or relapse, combining agents able to block essential signal transduction pathways with immunotherapy [12].

Earlier studies have identified specific mechanisms of BRAF inhibitor resistance in melanoma, which may reflect different temporal processes (early adaptive and late acquired resistance) [13, 14]. An example of the adaptive response is the upregulation of receptor tyrosine kinases with correlated AKT activation. Heterogeneous mechanisms of acquired BRAF inhibitor resistance in melanoma support the notion of MAPK and PI3K-AKT as two core resistance pathways. Key mechanisms include emergence of mutant BRAF-concurrent RAS or MEK mutations and mutant BRAF amplification or alternative splicing, but the relative contribution of non-genetic mechanisms to clinical disease progression is unknown. Distinct molecular lesions, in both core drug escape pathways, were commonly detected concurrently in the same tumor or among multiple tumors from the same patient. Beyond harboring extensively heterogeneous resistance mechanisms, melanoma re-growth emerging from BRAF inhibitor selection displayed branched evolution marked by altered mutational spectra/signatures and increased fitness. Thus, melanoma genomic heterogeneity contributes significantly to BRAF inhibitor treatment failure; co-targeting of two core pathways is an essential strategy for durable responses. Moreover, a clinical strategy to mitigate acquired BRAF inhibitor resistance by combined BRAF and MEK inhibition, although prolonging tumor suppression, is still beset by acquired drug resistance, suggesting MAPK-alternate escape routes. However, a genomic analysis of acquired double-drug resistance revealed a plethora of genetic alterations responsible for acquired single-drug (i.e., BRAF inhibitor) resistance. Further analysis uncovered that these genetic mechanisms often occurred in configurations indicating concurrent or exaggerated gene dosage alterations. A hallmark of acquired double-drug resistance in melanoma is augmentation of mutant BRAF gene dosage, e.g., V600E-BRAF ultra-amplification, mutant NRAS amplification, and homozygous loss of CDKN2A or PTEN. Examples of concurrent genetic alterations detected included: V600E-BRAF amplification + mutant MEK, V600E-BRAF amplification + DUSP4 loss and mutant NRAS + loss of PTEN. These genomic alteration patterns can result in profound alterations in the mode of signaling. For instance, in mediating MAPK pathway reactivation, up-expressed V600E BRAF dimerizes and activates CRAF or forms a complex with mutated BRAF; specific protein–protein interfaces were identified in these escape modes of MAPK pathway activation. Thus, even with the clinical superiority of combined BRAF/MEK inhibitors, melanoma nevertheless displays MAPK pathway addiction in acquiring resistance, suggesting that additional strategies to target the MAPK pathway are needed. Also, genomic analysis of acquired double-drug resistance does not readily identify accountable mechanisms in a large segment of clinical cases, suggesting a prevalence of non-genomic mechanisms. Lastly, acquired double-drug resistant melanoma clones were found to be highly addicted to the inhibitors, suggesting that drug addiction may be clinically viable strategy, through intermittent dosing, to suppress acquired resistance.

Inhibiting multiple pathways such as BRAF (vemurafenib or dabrafenib) and MEK (trametinib and cobemetinib) is a very promising and valuable approach in patients with MM. Combination BRAF/MEK inhibition could offer some advantages, despite of a median PFS of less than 10 months in one study. Double blocking strategy may be better than expected, producing tumor response in the majority of patients and sometimes durable CRs. Dabrafenib/trametinib combination therapy showed a prolonged OS benefit and greater response rate in patients with M1a/M1b disease and normal LDH values [15]. Both in the COMBI-V and COMBI-D randomized trials OS was significantly greater in the dabrafenib plus trametinib arm as compared with the vemurafenib or dabrafenib-only arm, with at least 30 % of death reduction rate favoring the combination arm [15–17]. Similar results have been reached in CoBRIM randomized trial [18].

Patients treated in the BRIM2 phase II study of vemurafenib in previously treated patients with BRAFV600E mutation and those treated with MEK-inhibitor cobimetinib + vemurafenib in the BRIM7 study have been evaluated for correlation between activity and intensity of MAP-Kinase inhibition [19]. When compared indirectly across the two studies, the combination of cobimetinib + vemurafenib resulted in enhanced inhibition of ERK phosphorylation and enhanced downregulation of transcriptional targets downstream of ERK at cycle 1 day 14 compared to vemurafenib monotherapy. The combination also inhibits pS6 signaling (82 %) and phosporylation of other mTORC-regulated proteins such as 4EBP1, eIF2a and eIF4G in BRAF-i naive patients. Targeting signaling initiated by NRAS and CDK4 are generating considerable interest and may lead to arrest of G1-S cycle cell progression or induction of senescence. Very promising drugs such as palbociclib a CDK4-inhibitor are being investigated preclinically and clinical investigation has commenced. In order to develop a new platform for drug development in MM the Melanoma International Collaboration for Adaptive Trials (MICAT) has been created. The goals of this study are to identify the most active combinations in advanced melanoma, identify the interaction with established and emerging biomarkers with the novel therapies, and to address key questions about sequencing strategy. BRAF regulates a complex network of transcription factors controlling glycolysis, involving MYC, MONDO-A and HIF1-alfa as final steps [20]. Rapid metabolic response is a typical feature following inhibition of BRAF and novel combinations are welcome to further enhance disease shrinkage. Another promising approach in mutated patients was the combination of BRAF-inhibitor with a PI3K inhibitor, which showed synergic activity in vitro [21]. Preliminary data of the combination of vemurafenib plus PX-866 showed durable ongoing responses in both treatment-naive and prior BRAFi or MEKi treated patients NCT01616199.

NRAS mutation is present in 15–20 % of all melanomas, characterized by poorer prognosis, with no targeted agents yet available. Nevertheless, these patients may respond better to immunotherapy, including high-dose interleukin 2 (IL-2) [22], and checkpoint inhibitors such as anti PD-1/PD-L1 drugs [23]. Binimetinib (MEK-162), an oral selective MEK 1/2 inhibitor, is currently under development together with other MEK-inhibitors, alone or in combination. MEK-162 has shown clinical activity in NRAS-mutated melanoma, with 63 % of disease control rate and 10 % of confirmed partial responses [24]. Combination of a MEK-inhibitor with a CDK4-inhibitor showed promissing results in patients with NRAS-mutated melanoma. The combination of oral selective inhibitors of MEK 1/2 (binimetinib) and CDK 4/6 (LEE011) has been tested in a phase IB/II study to block MAP-K signaling pathway at two downstream levels of RAS pathway [25]. Preliminary results on the first 21patients with MM showed an 86 % clinical benefit rate, often with early tumor shrinkage and major symptoms improvement. Patients remaining on study had an exposure to the study drugs ranging from 2 to 8 months. Common treatment-related adverse events included elevated serum CPK and creatinine, fatigue, skin, haematologic and gastrointestinal events. Combination treatment often required dose interruptions and reductions due to adverse events. The maximum tolerated dose (MTD) for the current dosing schedule was determined to be 200 mg/daily (3 weeks on-1 week off schedule) of LEE011 and 45 mg/bid (continuous schedule) for bimetinib. Exploration of intermittent schedules to improve tolerability and further analysis of effect of additional genetic alterations on clinical outcomes are underway.

The PI3K (phosphatidylinositol 3-kinase)-AKT pathway is a critical regulator of many essential physiological processes involved in cancer, including cell proliferation, apoptosis, motility, angiogenesis and metabolism. There is increasing evidence that activation of this pathway plays a significant role in melanoma, frequently in the setting of concurrent activation of RAS-RAF-MEK-ERK signaling. The PI3K-AKT pathway can be activated in multiple ways in melanoma, with the two most common being activating NRAS mutations and loss of the PTEN tumor suppressor. Melanomas with loss of PTEN have higher AKT activation than NRAS mutations [26]. In lymphadenectomy specimens from BRAFV600 mutation-positive patients with stage IIIB/C melanoma, complete loss of PTEN expression correlated with shorter time to brain metastasis (but not metastases to the liver, lung or bone) and decreased overall survival [27]. PTEN loss was prognostic and predictive of brain metastasis specifically in BRAF V600-mutant melanomas. Brain metastases have also shown increased expression of several activation-specific protein markers in the PI3K-AKT pathway compared with extracranial metastases, including in paired tumors from individual patients [28].

In BRAF-mutant human cutaneous melanoma cells treatment-induced activation of AKT has been shown to mediate resistance to cell death by the MEK inhibitor selumetinib (AZD6244) [29]. Inhibition of AKT activity, either by AKT knockdown or concurrent treatment with the mTORC1/2 inhibitor AZD8055, resulted in synergistic cell killing in selumetinib-resistant cell lines. In another study, a subset of BRAF- and NRAS-mutant human melanomas resistant to selumetinib were identified that were characterized by elevated oxidative phosphorylation (OxPhos), mediated by the transcriptional coactivator PGC1α [30]. Selumetinib-resistant high OxPhos cell lines, but not low OxPhos cell lines, could be resensitized to MEK inhibition by co-treatment with AZD8055. In both BRAF- and NRAS-mutant melanoma cells, MEK inhibition increased microphthalmia-associated transcription factor (MITF) expression, which in turn elevated levels of PGC1α. In contrast, mTORC1/2 inhibition resulted in cytoplasmic localization of MITF, thereby decreasing PGC1α expression and inhibiting OxPhos. Combined targeting of the MAPK and mTORC pathways may be a potential personalized therapeutic strategy for melanomas with increased OxPhos.

BRAF V600 inhibitors such as vemurafenib and dabrafenib leading to the activation, of several mechanisms of resistance [31]. Compensatory/adaptive responses to targeted inhibitors are frequently initiated by the activation of growth factor receptor tyrosine kinases, including ErbB3/Her3. ErbB3 is a potent activator of the PI3K/AKT pathway that expression correlates with melanoma progression. The receptor tyrosine-kinase (RTK) array profiling demonstrated hyperactivation of pErbB3 receptor in three different melanoma cell lines after treatment with vemurafenib. Enhanced pErbB3 signaling is accompanied by hyperactivation of pAKT and occurs also in cells exposed to a MEK inhibitor. These results suggest that enhanced ErbB3 signaling may serve as a mechanism of adaptive resistance to RAF and MEK inhibitors in melanoma. This feedback survival loop is promoted by increased autocrine production of neuregulin (NRG1) that is a ligand for ErbB3. Treatment with vemurafenib increases neuregulin gene expression in several cell lines. These findings support the idea that NRG1 acting in a paracrine manner, promotes resistance to RAF inhibitors and emphasize that targeting the ErbB3/ErbB2 pathway will likely improve the efficacy of RAF inhibitors for mutant BRAF melanoma patients.

The use of the anti-ERrbB3 monoclonal antibody (MAb) A4 abrogates the BRAF inhibitor-induced feedback loop and potentiates the vemurafenib inhibition on melanoma cells growth. ErbB3 mAb treatment impairs the resistance to vemurafenib in WM266 melanoma cells and restores drug sensitivity to vemurafenib including BRAFi-resistant cell lines [32]. The combination of two mAbs recognizing distinct epitopes on the ErbB2/Her2 receptor, has been shown to have a superior anti-tumor effect [33]. Similarly, the anti-ErbB3 A3 and A4 combination synergistically inhibits melanoma cells growth and accelerates ErbB3 targeting to lysosomal degradative pathway. It also abrogates both vemurafenib- and trametinib-induced ErbB3 activation and potentiates its inhibition of melanoma cells growth. A3/A4 mAbs combination synergizes with vemurafenib and trametinib in the inhibition of cell growth and in the induction of apoptosis, and strongly reduces tumor relapse in vivo after drug withdrawal.

The heat shock protein 90 (HSP90) family of chaperones maintains the malignant potential of cancer cells by regulating the conformation, stability and function of many receptor tyrosine kinases (RTKs) required for oncogenic transformation. Many proteins required for melanoma initiation and progression, including mutated BRAF, CRAF, CDK4 and AKT are known to be clients of HSP90 family members. This information has provided the rationale to use HSP90 inhibitors to overcome mechanisms of BRAF-inhibitor resistance. The glucose-regulated protein 94 (GRP94) is HSP90 like protein in the ER. GRP94-specific mAb W9 that recognizes an extracellular epitope of this protein and is able to overcome the BRAF-inhibitors resistance in human melanoma cell lines carrying a BRAF-V600E mutation. It increases the sensitivity of BRAF-mutant melanoma cells to BRAF-inhibitors and restores the sensitivity of resistant melanoma cells to BRAF-inhibitors. MAb W9 is expected not to cause significant side effects because it has limited or no reactivity towards normal tissues [34].

### Immunotherapy combinations

Before the immuno-targeted era, patients with MM experienced a median PFS at 6 months of 15 % and 1-year OS of 25 % [35]. However, in the immunotherapy era initiated with high-dose IL-2 therapy durable responses, some in patients with large volume and visceral disease [36], as well as in BRAF mutant and wild-type (WT) patients [22] were observed. Response was maintained in the off-treatment period, with 11 % of patients alive after 5 years from starting therapy. Ipilimumab showed a significant OS benefit in patients with either BRAF WT or mutant melanoma [37]. Moreover, BRAFi treatment was effective in patients with BRAF mutations who had received prior immune therapy [38, 39] but the proof of efficacy in the opposite sequence is not available. Because of this sequence variability BRAFi therapy may not be the best initial therapy for patients with BRAF mutation [40, 41]. A restrospective analysis showed that overall survival in patients who received ipilimumab followed by a BRAFi seems to be longer than in patients who received the treatment in reverse sequence, irrespective of the presence of prognostic adverse features such as elevated LDH and brain metastases [42]. However, prospective randomized data is not available to evaluated the two sequences. Current data suggest that for patients with BRAF mutation immunotherapy as initial treatment could offer long term benefit, without compromising the effect derived from subsequent BRAF-inhibitor therapy.

In the future, immunotherapy highly likely will be included in the therapeutic strategy in patients with BRAF-mutation, especially when better drugs and biomarkers for patients’ selection will become available. Newer drugs such as nivolumab (NIVO) are approaching the efficacy (ORR and PFS) of BRAF-inhibitors with more durability. Toxicity to BRAF-inhibitors, however, could be worse in patients who previously received immunotherapy. Severe adverse events were mainly dermatologic, with some cases reported of acute renal failure due to interstitial nephritis and of liver failure.

Combination of immunotherapy with targeted therapy may provide synergistic benefit in the treatment of MM, due to an improvement of long-term survival as compared to a single treatment. BRAF-inhibitors affect both the tumor and the immune cells in patients with melanoma. In the tumor, BRAFi have demonstrated potential to immunosensitize by up-regulation of melanoma differentiation antigens (MDA) expression and the CD8+ T cell infiltrate whereas, in the immune system BRAFi reduced the level of immunosuppressive cytokines and *increased PD-L1* expression [43]. When combining MEK-inhibitor with a BRAF-inhibitor MEK inhibitors may influence the cytokine production and immunosuppressive cell populations in the tumor microenvironment as observed in vitro [44]. MEKi trametinib also significantly improved the antitumor effect of BRAF inhibitor dabrafenib in combination with adoptive T cell transfer (ACT) and PD-1 blockade in BRAF V600E driven murine melanoma cells SM1 model [45]. Improved effector T cell homing to the tumors, increased MHC expression, cytokine release and attenuated immunosuppressive cells were observed in this study. These findings support testing these drugs in combination with immunotherapy in patients with BRAF-V600E mutated MM clinical trials. VEMUPLINT is a phase I–II study evaluating safety and efficacy of vemurafenib in combination with PEG-interferon in patients with BRAF-V600E mutation. Secondary objectives of this study are the IFN-alpha-receptor-1 (IFNAR-1) up-regulation and identification of markers of response to this treatment. Preclinical data also suggest the synergy of BRAF-inhibitors with anti-PD-1/PD-L1. MPDL3280A, a human Fc optimized anti-PD-L1 antibody (Roche), in combination with vemurafenib demonstrated promising activity in melanoma although with some grade 3 toxicity including rash and AST/ALT elevation [46]. Significant hepatotoxicity is also one of the major adverse events of vemurafenib in combination with anti-CTLA4 antibody ipilimumab [47]. In the phase I study combining dabrafenib + ipilimumab ± trametinib in patients with BRAF V600E/K mutation, the authors showed that standard doses of dabrafenib (150 mg bid) and ipilimumab (3 mg/Kg) can be administered without any severe grade toxicity and with all evaluable patients having a reduction in the sum of lesion diameter [48]. Patients treated with trametinib in addition to dabrafenib and ipilimumab had a greater incidence of gastrointestinal toxicity, with 2 patients having a grade 3 colitis and one patient with a grade 4 intestinal perforation [49].

Targeting the immune checkpoints (anti-CTLA-4, anti-PD1/PDL1) has a critical role both in the priming and in the effector phase of the antitumor immune response [50]. After becoming the first agent to demonstrate a significant OS improvement in a randomized phase 3 trial in metastatic melanoma [51], the anti-CTLA-4 antibody ipilimumab was approved for this indication. In addition to CTLA-4, more other immune check-points are potential targets for immunotherapy. For example, interaction of the programmed death 1 (PD-1) receptor with its ligands (PD-L1/B7-H1 and PD-L2/B7-DC) in peripheral sites leads to T-cell inactivation and loss of effector function. Targeting this pathway using antibodies against PD-1 (e.g., nivolumab, pembrolizumab) or PD-L1/PD-L2 prevents T-cell inactivation and restores immune activity directly at the tumour site [52]. Immunotherapies targeting other immune checkpoint molecules such as LAG3 are also under evaluation in advanced malignancies, either as monotherapy or in combination. Phase I nivolumab trial enrolled more than 100 patients with solid tumors, showing long-term overall survival benefit in all dose cohorts [53]. Patients with MM who received nivolumab 3.0 mg/kg q2w reached 20.3 months of median OS, with 32 % of patients who were alive at 4 years following treatment initiation. An open-label, phase III trial randomized patients with MM to receive nivolumab 3 mg/kg q2w or chemotherapy at investigator’s choice (dacarbazine or carboplatin + paclitaxel q3w) until progression or unacceptable toxicity. Patients were stratified according to PD-L1 expression, BRAF status and response to prior anti-CTLA4 therapy. Patients receiving nivolumab should be treated beyond progression if considered by the investigator to be experiencing clinical benefit and tolerating study drug. ORR, by central review, was threefold greater for nivolumab as compared with chemotherapy (32 vs. 11 %). In patients BRAF wild-type and treatment-naive nivolumab experienced, in a phase 3 randomized study, significantly greater OS (primary endpoint) as compared with dacarbazine [54]. One-year OS was 73 % in patients who received nivolumab and 42 % in those treated with chemotherapy (HR = 0.42—99 %CI 0.25–0.73; p < 0.0001). Response rate was more than doubled in patients treated with immunotherapy (40 vs. 14 %). In the phase I combination trial of nivolumab plus ipilimumab best results in terms of both efficacy and safety have been obtained with the IPI 3 mg/kg q3w for 4 doses + NIVO 1 mg/kg q3w for 8 doses schedule, followed by maintenance with NIVO 3 mg/kg q2w for no more than 48 doses [55]. In this cohort median duration of response has been yet not reached; 94 % of patients were alive at 1 year after treatment and 88 % at 2 years. IPI activity could also be enhanced adding the granulocyte/macrophage colony-stimulating factor (GM-CSF) sargramostim. In a randomized phase III trial the combination IPI + GM-CSF significantly increase median OS as compared with IPI alone [56]. The monotherapy arm has been characterized by more severe grade adverse events, especially gastrointestinal, probably due to a protective effect from sargramostim. A very important challenge is to identify factors predicting clinical response to anti-CTLA-4. Some authors have tried to select somatic mutations in patients with MM responding to IPI [11]. They found that mutational load was only associated but not predictive of response and identified a neoantigen panel which was predictive of response to IPI. PD-L1 status seems not to be a predictive factor because, in patients with MM who received a fully human anti-PD-1 mAb nivolumab (NIVO), OS was improved as compared to dacarbazine irrespective of PD-L1 status.

Treatment of MM with anti-PD-1/PD-L1 antibodies such as nivolumab or pembrolizumab resulted in 40 % ORR as first-line therapy and 20–30 % post-ipilimumab, with 65–75 % of patients still alive at 1 year. Possible mechanisms for innate resistance to PD-1/PD-L1 blockade are insufficient or disfunctional tumor antigen specific T-cells in tumor microenvironment, loss of tumor antigen presentation or innate tumor cell resistance to immune-mediated killing. Multiple combinations based on PD-1/PD-L1 blockade are supported by animal models, although no human data are yet able to predict the best combination for any individual patient. PD-1 and CTLA-4 have different roles in T-cells differentiation and regulation in the tumor microenvironment. For these reasons, the double blockade strategy seems to be more efficacious as compared to the single blockade in reducing median tumor volume. Phase I CA209-004 study evaluated, in different cohorts, the tolerability of nivolumab and ipilimumab given at different doses and as concurrent or sequencing schedule [55]. The cohort who reported the best efficacy/toxicity ratio was the sequencing schedule with nivolumab 1 mg/Kg + ipilimumab 3 mg/Kg every 3 weeks for 4 cycles followed by nivolumab 3 mg/Kg every 2 weeks for no more than 48 cycles. This schedule reported 44 % ORR (with 7 % CR) and 29 % of patients had at least 80 % reduction in tumor burden at 36 weeks. Responses were obtained in 50 % of patients with BRAF mutations and in 37 % with wild-type status. Almost 2/3 of patients in this cohort experienced at least one grade 3–4 adverse event, mainly rash, diarrhea and ALT or lipase increase. Treatment-related, immune-mediated, severe grade adverse events in this cohort were mainly skin (17 %), gastrointestinal (20 %) and hepatic (12 %). Questions to be addressed about the anti-PD-1/PD-L1+ anti-CTLA4 combination therapy concern what is the best schedule (concurrent vs. sequential), the management of adverse events, the search for predictive biomarkers and the safety of triple combinations. However, if phase III trials will confirm early data, the combination of an anti-PD-1+ an anti-CTLA-4 could become standard of care in patients with MM, in both BRAF WT or mutated status.

Use of vaccines that stimulate strong immune responses against the specific targets in combination with drugs that can inhibit the immune-suppression of T-cells could be one of the promising strategies to optimize immunotherapy in MM. Drugs such as anti-PD-1 antibodies and low-dose chemotherapy with cyclophosphamide (LD-CPM) that targets T regulatory (Treg) cells with minimal effect on other T-cell populations [57] could be the optimal candidates for the combination therapies. Further, anti-PD1 antibodies and LD-CPM are able to synergistically decrease and maintain low level of Tregs infiltration in the tumor and in the periphery. Addition of the vaccine to this combination enhances the level of tumor-infiltrated CD8+ T-cells and of CD8/Tregs ratio in tumor microenvironment thereby promoting tumor rejection. Clinical phase II trials utilizing this combination of anti-PD1, LD-CPM and vaccines are currently undergoing in prostate and pancreatic cancers as adjuvant therapy.

PD-1 can also be targeted using a recombinant protein, B7-DC-Ig. This is composed of an extracellular domain of murine B7-DC, a PD-1 ligand fused to Fc portion of murine IgG2a. B7-DC-Ig has been shown to enhance the therapeutic efficacy of vaccine when combined with cyclophosphamide. The combination significantly enhanced the Ag-specific immune responses, leading to the complete eradication of established tumors in 60 % of mice and the effect was observed to be CD8-dependent [58]. It is also important to determine the optimal scheduling of various immunomodulators in combination therapies. For example, B7-DC-Ig was more effective when administered in a sequential schedule with cyclophosphamide and vaccine as compared to a simultaneous schedule in a CD4-dependent model. Further, B7-DC-Ig showed an antitumor effect only when administered at the end of the schedule. Similarly, anti-PD-1 exhibits prolonged survival only when given together with vaccine.

In addition to inhibitory strategies, T-cells stimulators such as OX40 can also be used in combination therapies for cancer. OX40 (CD134) is a tumor-necrosis factor receptor (TNFR) that is mostly expressed on activated T-cells (both CD4+ and CD8+) and transmits a potent co-stimulatory signal when activated. OX40 enhances T-cell responses and is associated with increased T-cell expansion and proliferation, survival, and memory development. Anti-OX40 antibody is a T-cell stimulator, which can be considered as a promising anticancer approach in combination with vaccines. In fact, treatment of tumor bearing mice with an anti-OX40 agonist antibody in combination with a specific vaccine showed an enhancement of antigen-specific T-cells responses [59].

Tumor cells are able to produce a number of suppressive signals as a protective mechanism against their destruction, such as increased expression of indoleamine-(2,3)-dioxygenase (IDO). IDO is an important enzyme in the tryptophan metabolism and is shown to downregulate activating natural killer cell receptors on NK cells and also to inhibit cytotoxic T cells (CTL) [60]. Therefore, it has been targeted with an inhibitor, 1-methyltryptophan (1-MT) for cancer therapy.

In conclusion, combination therapies that target immune-inhibitory and immune-suppressive molecules as well as stimulate immune-stimulatory molecules have been developed and investigated. The biological effects of combination immune therapies are, however, dependent on several factors such as the biology and mechanisms, dose, sequence, and timing of the specific agents as well as the type of effector response, which need to be carefully considered for optimum therapeutic effects.

### Other combination approaches

Ionizing radiations have pro-immunogenic effects at multiple levels. Immunostimulation achieved by combining immunotherapy with localized radiation can lead to the so-called abscopal effect, consisting of tumor response outside the radiation field. Patients with an abscopal response are more likely to be already more immunocompetent, with preclinical and clinical data supporting this feature because abscopal effect seems to be abrogated in nude mice [61] and in patients with high ratio of circulating neutrophils/lymphocytes [62]. The hypothesis which supports the synergistic effect of radiotherapy in combination with immunotherapy is that ionizing radiations can stimulate anti-tumor immunity by generating an in situ vaccine, and combination with immunotherapy may enhance this effect.

Radiation therapy (RT) also modifies the immune system interaction with cancer, driving the tumoral tissue from a lymphocyte-poor to a lymphocyte-enriched environment [63]. In part, this effect is the result of RT upregulation of chemokines, seen in multiple mouse and human cancer cells, which enhance CD8+ T-lymphocytes recruitment and tumor infiltration [64]. The RT also upregulates retinoic acid early inducible-1 (RAE-1), a ligand for natural killer (NK) cell group 2D (NKG2D) receptor (RAE-1). RAE-1 stabilizes the immune synapses between CD8+ T-lymphocytes and target tumor cells promoting the killing activity of anti-CTLA-4 antibody-activated T cells [65].

Pre-clinical evidence shows that the magnitude of anti-tumor CD8 T cell responses activated by the combination of RT and anti-CTLA-4 treatment is dependent on the availability of dendritic cells (DCs) in the tumor and draining lymph nodes. In a mouse model of triple negative breast cancer type I or invariant NK (iNKT) cells, which are CD1d-restricted T-lymphocytes recognizing lipid antigens presented by DCs, regulate DCs numbers limiting the efficacy of treatment. INKT-deficient mice have significantly more DCs in the tumor and draining lymph nodes and show evidence of some spontaneous anti-tumor immunity. Response to treatment with CTLA-4 blockade and RT is markedly improved in mice lacking iNKT cells or treated with an antibody blocking their interaction with DCs by binding to CD1d [66, 67].

Another critical factor that determines the efficacy of RT with anti-CTLA-4 antibody is the radiation regimen employed. While radiation-induced immunogenic cell death in vitro is dose-dependent [68], in vivo three to five fractions of 6 or 8 Gy are more effective than a single 20 Gy dose in inducing immune-mediated rejection of the irradiated tumor and non-irradiated synchronous tumor in combination with anti-CTLA-4 [69].

Confirming results obtained in preclinical studies [70], addition of radiation to one tumor site was shown to induce systemic responses (abscopal effects) in patients with melanoma resistant to anti-CTLA-4 treatment alone [71]. A similar synergy of radiation with anti-CTLA-4 was reported in a patient with lung cancer, a tumor type previously shown to be unresponsive to single agent anti-CTLA-4 [72]. Multiple clinical trials testing the activity of RT in combination with ipilimumab in solid tumors are ongoing. Results of a randomized, double-blind, placebo-controlled phase III study of ipilimumab plus palliative bone RT (8 Gy in single fraction) in patients with metastatic, castration-resistant, prostate cancer who have progressed after docetaxel have been recently published [73]. The study was powered to detect a 4-month difference in median overall survival between the two arms but failed to meet its primary endpoint. Survival curves initially favored the placebo arm but, after 6 months, they split showing an evident advantage for patients who received ipilimumab. In the experimental arm, the 6-months PFS was 30.7 % as compared with 18.1 % in the placebo arm. Possibly, factors like the inclusion of patients with visceral metastasis, the choice of bone metastasis as radiation target, and a single dose fractionation may explain the limited success of the combination in this trial [74].

Other immune response modifiers have shown synergy with RT. Imiquimod (IMQ) is a synthetic toll-like receptor 7 (TLR7) agonist approved in the US as a topical cream for treatment of superficial basal cell carcinoma, external genital warts and actinic keratosis. In the priming phase, the activation of TLR7 leads to the activation of T-helper-1, B lymphocytes and natural killer cells. IMQ has shown some activity in patients with cutaneous breast cancer metastases [75, 76]. In preclinical studies, the combination of topical IMQ and RT was more effective than each agent used alone and induced abscopal effects [75]. A clinical trial is currently ongoing to test this combination in the clinic.

The anti-TGF-beta antibody fresolimumab has also been tested in combination with RT in metastatic breast cancer (NCT01401062). Median OS and PFS appeared to be better in patients treated with fresolimumab at 10 mg/dose as compared with those who received 1 mg/dose. Preclinical studies demonstrate that TGF-β, which is activated by RT, is a dominant immunosuppressive barrier precluding the generation of an effective in situ tumor vaccination by RT [77].

Overall, preclinical and clinical evidence suggests that local radiotherapy can help the efficacy of cancer immunotherapy, by rendering the irradiated tumor more immunogenic. Radiotherapy can be harnessed as an adjuvant to immunotherapy as it may convert non-responders patients to responders to the same immunotherapy. Dose fractionation and sequencing of radiotherapy need to be explored in combination with each immunotherapy strategies, in prospective clinical trials. Many questions remain to be addressed in this field, regarding the optimal sequencing of radiotherapy and immunotherapy, the best dosing and fractionation schedule and patients selection. There is also a lack of data about the optimal site to be irradiated in metastatic disease, in order to obtain the best immunological response.

Tumor vaccines that consist of strategies that intend to prime and boost specific immune responses in a tumor bearing host can be combined with immunostimulatory mAb in clinical trials [78]. Virotherapy has been developed in gene therapy, using viruses as vectors for genes encoding vaccines and antitumoral transgenes. Semliki Forest Virus (SMV) has been used as a vector to express IL-12, which than can be injected into subcutaneous tumors-bearing mice. In melanoma model, the antitumor activity of SFV-IL-12 vector given intratumorally can be enhanced adding an agonist antibody to CD137 costimulatory receptor on T cells given systemically [79]. This is also true with anti-PD-1 mAbs [80]. CD8+ cells are the main mediators of the curative response. This efficacious combinatorial immunotherapy strategy offers feasibility for clinical translation since anti-CD137 mAbs are already undergoing clinical trials and development of clinical-grade SFV-IL-12 vectors is in progress.

According to the final report of the study comparing sentinel-node (SN) biopsy vs. nodal observation in patients with melanoma, SN provide very important prognostic information and help to identify patients with nodal metastasis whose survival can be prolonged by immediate lymphadenectomy [81]. SN biopsy improves overall survival as compared to observation (HR = 0.54—95 % CI 0.37–0.80, p = 0.0017). The number of excised lymph nodes seems to be very important in determining melanoma-specific survival. A retrospective review of 2536 patients showed the best survival in patients with more than 30 lymph nodes removed and the worse in those with less than 10 lymph nodes excised [82]. No difference on survival has been detected in patients with 10–30 lymph nodes excised. Multiple single institution series indicate long-term survival (22–23 % at 5 years) following resection of multiple distant metastases for melanoma [83–87]. Prognostic factors in surgically resected stage IV melanoma have been identified in clinical trials [88–91] including:

- ability to achieve complete surgical resection of all visible disease
- tumor-doubling time
- number of metastatic lesions (solitary vs. multiple)
- disease-free interval (>36 vs. ≤36 months)
- site of recurrence (brain vs. liver vs. lung or regional lymph nodes)

Electrochemotherapy (ECT) allows the permeation into cancer cells of poorly permeable antineoplastic drugs, such as bleomycin or cisplatin that allow for optimal concentration in the tumor tissue. ECT could be useful in treating cutaneous melanoma with curative intent or as neoadjuvant therapy to obtain shrinkage of the tumor. ECT may thus be used in the management of the tumor and less invasive surgery. Multiple treatments are possible, with about 75 % of overall response rate and 21.7 % of long-lasting response [92]. Electrochemotherapy seems also to be effective in combination with ipilimumab (IPI), with 60 % of disease control rate and a decreased Treg values in responders patients [93].

## News in immunotherapy

Tumor antigen-specific CD8+ T cells in the periphery and at the tumor site co-express a number of inhibitory receptors including PD-1, BTLA and Tim-3. These inhibitory receptors are upregulated upon T cell activation and are often co-expressed by dysfunctional CD8+ T cells in the periphery and at tumor sites. We have observed that tumor antigen-specific CD8+ T cells co-expressing PD-1 and Tim-3 are highly dysfunctional in terms of proliferation and cytokine production while PD-1^+^BTLA^+^ Tim-3^−^ CD8+ T cells appear less dysfunctional [94, 95]. In sharp contrast, we also observed that PD-1^+^ BTLA^−^ Tim-3^−^ tumor antigen-specific CD8+ T cells are functional T cells, supporting that PD-1 is an activation marker that is also upregulated together with other inhibitory receptors by tumor antigen-specific CD8+ T cells upon chronic antigen activation. Interestingly, dual PD-1/BTLA and PD-1/Tim-3 blockades were superior to each single blockade in enhancing the expansion and function of tumor antigen-specific CD8+ T cells in vitro. Altogether, our findings support the use of dual PD-1/BTLA blockade and dual PD-1/Tim-3 blockade to further augment the expansion and functions of spontaneous CD8+ T cell responses to tumor antigens in patients with advanced melanoma.

In the context of a vaccine trial with CPG and MHC class I and class II peptides from the tumor antigen NY-ESO-1, we observed that patients with advanced melanoma immunized with CPG, MHC class I and MHC class II epitopes developed higher frequencies of vaccine-induced CD8+ T cells with lytic capacities as compared to patients immunized with CPG and MHC class I peptide alone [96]. Interestingly, vaccine-induced CD8+ T cells upregulated PD-1 in their large majority while a minority co-expressed PD-1 and Tim-3. We also show that dual PD-1/Tim-3 blockade further enhanced the expansion and functions of the vaccine-induced CD8+ T cells in vitro. These data strongly support combinatorial therapies with cancer vaccines and PD-1 blockade to prime and expand potent CD8+ T cell responses in patients with advanced melanoma. Such approach may be beneficial to melanoma patients with no/poor spontaneous T cell responses to melanoma and who may be less likely to respond to PD-1 blockade alone.

Another target of interest in the tumor microenvironment is the IL-10/IL-10R pathway. IL-10 receptor (IL10R) is a marker of T cell activation that is upregulated by PD-1^high^ CD8+ TILs and by tumor antigen-specific CD8+ T cells upon PD-1 blockade [97]. We observed that IL-10 acts directly on T cells to induce the apoptosis of PD-1^high^ tumor antigen-specific CD8+ T cells. Dual IL-10R/PD-1 blockade enhanced the expansion and functions of tumor antigen-specific CD8+ T cells in patients with advanced melanoma. Such combinatorial approach warrants further exploration in the clinic.

Tumor-associated antigen such as MAGE-A1 and mutated CDK4 identified by the “classical” method of CTL-guided molecular cloning are well known in melanoma. Deep sequencing has revealed that melanoma displays a very high frequency of non-synonymous somatic mutations. CD8 T cell reactivity against patient’s-specific somatically mutated antigens have been recently reported, in the context of immune checkpoint blockade therapy [98]. The CTL/Treg ratio is an important factor in determining the success of tumor immune responses and is an independent prognostic factor [99]. The role of the antigen-specific effector T cells (Teff)/Treg ratio, its functional outcome, and the potential of manipulating this balance by vaccination remains understudied. Some authors have tried to determine the role played by the type of adjuvant vaccine in modulating the tumor antigen-specific Teff/Treg response. Teff accumulation among TIL depends on the adjuvant vaccine used and correlates with tumor protection. Moreover, the Teff/Treg ratio in tumor-draining lymph nodes correlate with CTL infiltration in the TIL in the tumor site and enhanced the tumor growth control. Tumor vaccines such as CpG and Poly(I:C) induce inflammation and a rapid production of Th1 cytokines in the vaccine draining LN. They also dramatically increase the antigen-specific Teff/Treg ratio in the lymphoid organs, skewing the immune response in favour of a functional anti-tumor effect [100]. This study suggests that choice of the adjuvant is important for peptide vaccine efficacy. Also, that Teff:Treg ratio can serve as biomarekrs predicting the vaccination outcome.

Moreover, micro-RNA expression profiling identified a limited set of differentially expressed microRNAs including miR-21, miR-17 to 92, miR-146a/b and miR-155 in primary human (and murine) CD8 T cells, suggesting a role in CD8 differentiation and/or function [101]. miR-155 is upregulated in vivo in differentiated mouse CD8 subsets, promoting CD8-T cells proliferation [102]. miR-155 expression is necessary for efficient CD8 T cell responses to virus, vaccination and cancer and is important for accumulation but not differentiation of effector CD8 T cells. Of note, miR-155 deficient mice have impaired memory CD8 T cells and the mechanisms leading to this impairment remain to be identified. Preliminary results suggest that miR-155 expression levels are higher in the tumour infiltrated tissues than in non infiltrated ones in malignant melanoma cancer patients. In addition, miRNA-155 overexpression in tumor-specific CD8+ T cells substantially increased their potency (Martínez and Romero unpublished), thus providing strong evidence for a clinical potential in the context of therapeutic adoptive T cell transfer.

One of the most common immune escape strategies carried out by tumor cells is altered signaling at different levels or immune response mechanism. Loss or down-regulation of HLA antigens has been frequently observed in melanoma cells with increased frequency of deficiencies in metastatic cells vs. primary lesions. Alterations in the tumor cell surface expression of the HLA class I antigens are often associated with the ability of cancer cells to escape from the antitumor immune response. Impaired HLA antigen expression is associated with a worse prognosis and reduced survival of patients.

There are frequent aberrations in the expression of components of the antigen-presenting machinery in melanoma lesions as a result of down regulation at the transcriptional, epigenetic and posttranscriptional level or structural alterations, including deletions/mutations and polymorphisms. Micro-RNAs (miRs) are about 22 nt long non-coding RNAs that are key mediators of post-transcriptional processing. In tumors, they control many processes such as apoptosis, proliferation, migration, immune responses and are associated with neoplastic transformation, disease progression and poor patients’ outcome. Transporter Associated with Antigen Processing 1 (TAP1) is an HLA class I processing machinery component that is regulated or even directly targeted by specific miRs species in tumor cell lines including melanoma. Another feature is also an impaired response to IFN due to a link between the IFN signaling and the HLA expression. Different mechanisms of IFN resistance as well as the induction of an immune tolerance could be demonstrated in melanoma cells. JAK-2 kinase gene deletion on chromosome 9 has often been reported in IFN-γ resistant melanoma cells. JAK-2 deficient melanoma cells have a reduced expression of the antigen-presenting system components, a mechanism that can be reversed with JAK-2 overexpression [103]. Similar results have been shown for STAT-1 (unpublished data).

Adoptive cell therapy (ACT) is an effective treatment for patients with metastatic melanoma. Adoptive cell therapy with tumor infiltrating lymphocytes (TILs) using IL-2 shows objective responses in about 50 % of patients in clinical trials [104], with a 20 % of complete responses. In a series of three consecutive clinical trials using increasing lymphodepletion before infusion of autologous TILs, objective response rates between 49 and 72 % were seen. Responses occur at all sites and appear to be durable with many patients in ongoing response beyond 3 years. Mean telomere lenght, the number of CD27+/CD8+ cells and the persistence of the infused cells in peripheral blood at 1 month after cell infusion are significantly different in objective responders (CR + PR) as compared with non-responders [105]. In another trial using TILs and non-myeloablative lymphocyte-depleting chemotherapy in patients with MM, a shorter duration of expansion and infusion of more CD8+ cells is associated with better clinical outcomes [106]. TILs from responder patients also contain more CD8+ cells, while quantities of CD4+ cells did not affect clinical outcomes [107]. Some efforts are ongoing in order to produce more potent TILs or to expand their intratumoral infiltration in patients with MM. Examples are the co-stimulation through the 4-1BB/CD137 antibody, which increases the CD8+ T cells frequency [108], and the use of K562-derived artificial APCs, which acta s feeder cells for T-lymphocytes expansion [109].

Among methods to improve adoptive T-cell therapy following non-myeloablative chemotherapy, the administration of checkpoint inhibitory antibodies activating T cells or depletion of MDSCs in order to reduce immunosuppression are proposed. Another method is re-stimulation of the injected TILs with a tumor vaccine (e.g., dendritic cells based) expressing the same antigen as recognized by TILs to improve life-span of the antigen specific T cells. MAT-02 is an ongoing clinical trial using the approach of combining TIL infusion with vaccination of autologous-lysate-loaded DCs, an approach which in an earlier pilot trial MAT-01 was feasible and demonstrated some clinical responses [110]. MDSCs in different cancer types are associated with different phenotypes (granulocytic or monocytic). Monocytic cells CD14 + HLA-DR- MDSC (so-called mo-MDSC) numbers are greatly increased in melanoma patients and strongly suppressive of T-cells proliferation and IFN-γ production [111]. Mo-MDSC are also suppressive for NK cells activity and can impair the quality of dendritic cells [112, 113]. Studies of the mechanisms involved in MDSCs induction and suppressive activity in melanoma patients is ongoing. MDSCs represent potentially a new target for cancer immunotherapy, especially in melanoma. Other strategies to counterbalance tumor-driven immune dysfunction are reversing the oxidative stress in cancer patients by the administration of histamine and antioxidants (e.g., vitamin E), and arming T cells by gene transfer of antioxidants enzymes such as catalase or thioredoxin [114]. In breast cancer, T cells transduced with a HER2 specific CAR-catalase can kill HER-2 positive breast cancer cells more efficiently while under oxidative stress conditions (Ligtenberg et al. submitted). Checkpoint specific antibodies blocking CTLA-4 or PD-1/PD-L1 affect both monocytic and granulocytic MDSC’s. Monitoring the immune system during treatment with ipilimumab, showed a reduction in frequency of granulocytic MDSCs and Tregs, often after just one dose, both in responders and non-responders patients [115]. The predictive value of the change in decrease of these cell subsets for the therapy outcome, however, remains to be determined.

A particular kind of T cell, expressing the so-called Chimeric Antigen Receptor (CAR) and directed to CD19, have demonstrated very high activation ligand affinity and are very effective in treating hematological tumors such as acute lymphocytic leukemia [116] and B cell lymphoma [117]. Anti-CD19 CAR T cells remain in the circulation for approximately 28 days and greater quantities of circulating CAR T cells are associated with better clinical responses. However, treatment with CD19 CAR T cells has been associated with a peculiar toxicity profile which is characterized by tumor lysis syndrome, cytokine-release syndrome (CRS) and neurotoxicity. Cytokine-release syndrome is a potentially life-threatening toxicity which can be observed following administration of antibodies and adoptive T-cell therapies for cancer. CRS is typically associated with high circulating levels of cytokines such as IL-6 and IFN-gamma, and can be managed with corticosteroids or the use of tocilizumab, an anti-IL-6 antibody. The use of tocilizumab and steroids should be limited only to the potentially life-threatening cases, since they may limit the effectiveness of the immunotherapy [118]. CRS is more likely to occur in patients with greater tumor burden and greater quantities of circulating CAR T cells. High serum level of IL-6, IFN-gamma and C-reactive protein are associated with more severe toxicity.

## Tumor microenvironment and biomarker

In 1863, Virchow first described leukocytes in malignant tumors, believed to be “cells of origin” of cancer. Tumor-infiltrating lymphocytes (TILs) have been cited by Ehrlich (in 1909) as host response to tumor. More recently, TILs have been associated with favorable prognosis in cancer patients, acting as a background for modern therapies such as targeted therapy and immunotherapy [119]. A widespread TILs diffusion in primary melanoma is associated with prolonged survival and reduced risk of metastasis. In lymph node metastasis, high amount of TILs improve survival as compared to lesions with low-to-absent TILs infiltrates. Radial growth phase of primary melanomas commonly show lymphocytes that can cause partial tumor elimination. Cytolytic immune responses against autologous tumors in melanoma patients support the idea that TILs play a key role in immunologic tumor clearance. Tumor-reactive cytotoxic T lymphocytes in the blood, lymph nodes, and in lymphocytes infiltrate primary tumors and metastatic nodules of many cancer patients. Clonal CTL expansions have been identified in primary and metastatic melanomas undergoing spontaneous regression. These immune effector populations were found capable of cytotoxicity against autologous cancer cells. Adoptive transfer of autologous TILs in combination with IL-2 treatment resulted in tumor regression in metastatic melanoma. Prolonged survival by TILs have also been demonstrated in other cancers such as ovarian and colorectal.

The classic approach to TILs as a prognostic indicator utilized the “brisk/non-brisk” system, developed by Clark [120]. “Brisk” indicates when the entire base of the tumor is infiltrated by TILs (peripheral) or when TILs diffusely meet the tumor (diffuse). “Non-brisk” is when isolated, multifocal and segmental TILs infiltrate the tumor. “Absent” is when no lymphocytes are directly apposed to tumor cells. In a retrospective series of 285 cutaneous melanomas, “brisk” feature was associated with the best overall survival as compared with “non-brisk” or “absent” TILs. 5-years survival rate was 77 % in patients with “brisk” infiltration as compared with, respectively, 53 and 37 % in “non-brisk” and “absent”. These results have been confirmed by preliminary data from the EORTC trial, involving 1260 patients with advanced melanoma and still under further statistical analysis by Dr. Phyllis Gimotty (University of Pennsylvania). The “brisk” peripheral pattern was associated with the best prognosis, especially in lesions >4 mm thickness, while the “non-brisk” peripheral pattern had the worse outcome. Impact of TILs on survival appears to be greater when there is no ulceration in the melanomatous primary lesion. TILs density can be scored according to the number of lymphocytes detectable per high power field (HPF): grade 1 (no more than 10 × HPF), grade 2 (11–20 × HPF) and grade 3 (more than 20 × HPF). Patients with grade 3 TILs density are associated with better overall survival as compared to the lower scores [1]. The “brisk” peripheral pattern is also known as the advancing edge of the tumor, and the EORTC study emphasizes its significance as a prognostic factor.

Three other studies support the importance of the advancing edge. Phospho-ERK (pERK) and Ki67 expression seem to be good prognostic factors in patients with stage I/II melanomas. pERK low (less than 20 %) and Ki67 low (score less than 94) phenotype has better survival and time to development of metastasis as compared with pERK and Ki67 high scores. SOX2-positive melanomas were significantly associated with a greater likelihood (twice as likely on average) of being 1 mm thicker than their SOX2-negative comparator. SOX2 expression correlated with primary tumor thickness in survey cohort (OR = 2.01 [1.04, 3.92], p = 0.039). SOX2 positive cells in patient melanomas are concentrated at the interface of tumors and the surrounding stroma and are observed in association with peritumoral vessels. SOX2 knockdown impairs melanoma cell invasion and co-localize with MMP-3 at infiltrative borders of patient melanomas [121]. Tumorigenic melanoma cells in spheroids or nodules strongly and diffusely express nestin. In infiltrative tumor cells at margin, the nestin is restricted to the subplasma membrane region of the cell. This distribution in human melanoma cells and experimental animals is associated with EMT with fusiform cell shape, increase in MMP, invasion, and multiple FAK attachment sites [122].

In most studies, there are tumors that do not show any response to TILs. A possible explanation for this immune evasion include Tregs failure. Tregs are 5–10 % of murine and human CD4+ T cell. They suppress CD4+, CD8+ T-cell, and NK cell responses and negatively regulate immune responses in vivo. Tregs deficiency is associated with massive T cell lymphoproliferation and multi-organ autoimmune disease (IPEX-human), characterized by immune dysregulation, polyendocrinopathy, enteropathy, and X-linked inheritance. Percentage of Tregs in patients with epithelial malignancies is more than doubled as compared with patients without tumors. Tregs removal also evokes an effective anti-tumor immunity in experimental models.

In conclusion, TILs apparently play an important role in determining the progression of malignant melanoma. In many situations, however, TILs appear to be ineffective. Manipulation of the immune system, based on understanding of T cell function and natural immune checkpoints gives great hope for further improve survival in malignant melanoma.

Lymphatic mapping and sentinel node biopsy (LM/SNB) for melanoma was first described 30 years ago. Two major worldwide clinical trials have been undertaken. MSLT1 enrolled 2001 patients who were randomly assigned to wide excision and nodal observation, with lymphadenectomy for nodal relapse (observation group), or wide excision and sentinel-node biopsy, with immediate lymphadenectomy if nodal metastases were detected on biopsy (biopsy group). MSLT1 has been completed and definitive 10-years follow-up results were published last year [81]. Biopsy-based management improved the 10-year rate of distant disease-free survival (hazard ratio for distant metastasis, 0.62; p = 0.02) and the 10-year rate of melanoma-specific survival (hazard ratio for death from melanoma, 0.56; p = 0.006) for patients with intermediate-thickness melanomas and nodal metastases. LM/SNB accurately stages regional nodes (95–97 % accuracy), identifies patients with clinically occult metastatic disease in their sentinel node who are candidates for immediate completion lymph-node dissection (CLND). During clinical observation small metastases in SN become detectable and may spread to additional nodes (3.2 vs. 1.4, p = 0.001); SN metastases may also spread to distant sites (HR = 0.62—p = 0.0152). The early lymph-node surgery approach is associated with substantially less morbidity.

Although CLND is standard for patients with SNB metastases, MSLT1 data show that 88 % of patients with a single tumor-containing sentinel node will have no additional nodal metastases when the CLND specimen is examined by hematoxylin-eosin staining. If nodal metastases are limited to one or two sentinel nodes, SNB might be therapeutic as well as prognostic. The MSLT2 trial was designed to examine this possibility. The underlying hypothesis is that CLND can be avoided in most patients with sentinel node metastases. MSLT2 has completed accrual and 10-year follow-up data are due in 2019. The MSLT trials helped identify two major primary melanoma subgroups, with differing risks of developing nodal metastasis. The major risk of node metastasis is affects tumors having ≥1 mm Breslow thickness or <1 mm with mitoses or ulceration. These patients should be considered for sentinel node biopsy. Patients with melanomas <1 mm Breslow thickness and neither mitoses or ulceration have a low-to-absent risk of nodal metastases. They can receive more conservative treatment, based on wide excision and clinical follow up. After SN biopsy, about 80 % of nodes are microscopically negative: such patients can be considered as optimally staged and receive follow-up only. False negativity of SN accounts for less than 5 % in experienced hands. The 20 % of patients with microscopically positive SN have to be considered for immediate CLND. However, since more than 80 % of patients treated by immediate lymphadenectomy showed tumor-negative non-sentinel nodes, surgical overtreatment is a concern. To identify patients receiving unnecessary CLND, authors have evaluated parameters such as the number of SN with metastases, the site, size and frequency of metastases and the percentage of node replaced by tumor. They also performed a molecular pathology assessment with gene expression microarrays (GEM), in order to develop potentially predictive gene signatures. They found that the amount and disposition of tumor in the SN can predict groups of patients at risk for non-SN tumor, tumor recurrence and death from melanoma. Main survival predictors were the sentinel node tumor burden (p = 0.0023), tumor diameter (p = 0.0173), tumor depth of invasion from capsule (p = 0.0096), the number of metastatic foci (p = 0.0063) and extra-capsular extension (p = 0.0148). Molecular pathology assessment showed significantly different gene expression profiles comparing SN metastases from patients with and without metastasis in non-SN. Authors developed two preliminary gene signatures: signature 1 contains 22 over-expressed genes related to cell cycle control, adhesion and proliferation, which are up-regulated in patients with metastasis-positive non-SN. In the signature 2 there are 22 genes related to host immune functions (activation, maturation of T, B lymphocytes and dendritic cells), which appear to be under-expressed and down-regulated in patients with metastasis-positive non-SN. These gene signatures, although preliminary, will likely enhance the accuracy of prediction of non-SN tumor status, identifying patients likely to benefit from CLND. This will spare many patients the morbidity of extended surgery and significantly reduce medical costs. More accurate staging will also identify patients at high risk of visceral metastases who may benefit from relatively aggressive adjuvant therapy.

There are three major hypotheses to explain the molecular mechanisms which explain the T cell-inflamed vs. non-T cell-inflamed tumor microenvironments. Germline genetic differences are hypothesized to occur at the host level, such as polymorphisms in immune-regulatory genes, that could set thresholds for activation of immune cells. At the tumor cell level, somatic differences leading to activation of distinct oncogene pathways vary between, and specific pathways could lead to T cell exclusion. The Wnt/β-catenin pathway has been identified as the first pathway that mediates immune exclusion and resistance to immunotherapies [123]. Preclinical data have also supported a role for the commensal microbiota impacting on systemic anti-tumor immunity, and specific commensal bacteria have been identified that can facilitate immune-mediated tumor control as a pro-biotic. Identifying these factors is important, as T cell-inflamed tumor microenvironment may act as a predictive biomarker for response to immunotherapies. Most immunotherapy responders (vaccines, anti-CTLA-4, anti-PD-1) have a microenvironment characterized by high production of chemokines and a CD8^+^ T-cell infiltrate. The mechanism of generation of a spontaneous anti-tumor immune response appears to be driven by the host STING/IFN- β pathway, activated by tumor- derived DNA acquisition by dendritic cells [124]. STING agonists may provide means to deliberately initiate innate immune inflammation to promote an endogenous T cell response in non-T cell-inflamed tumors. In multiple tumor models, intratumoral injection of STING agonists promoted strong endogenous immune responses and triggered durable tumor rejections [125]. The host STING pathway is also necessary to obtain a therapeutic effect from radiation therapy in vivo [126], providing another means by which anti-tumor immunity can be facilitated.

Recently, the importance of the adaptive immune reaction, in terms of nature, functional orientation, density and location within different tumor regions, as a counterpart of tumor invasion has been recognized [127, 128]. However, the development of a simple and powerful clinical test (“immunoscore”) based on the complex intratumoral immune reaction has been a difficult challenge [129]. The immunoscore displays characteristics related to the adaptive immune response, with regard to the cell type (CD3+ and CD8+ T cells) as well as their density and location (intratumoral vs. margins) [130]. In order to be implemented into the human cancer classification and patients management i.e., determine patients risk and assign patient to specific therapy including immunotherapy, respectively, the “immunoscore” needs to be analytically validated and undergo clinical validation process. So far the value of the immunoscore has been well established in patients with early-stage (I–II) colorectal cancer where the immune profile seems have higher prognostic importance than clinical features such as TNM-staging system [131]. The process for immunoscore validation is currently ongoing worldwide using a standard operating procedure and an assay harmonization involving 23 centers in 17 countries. Prospective observational trial for clinical validation of the prognostic value of the immunoscore is currently ongoing (NCT01688232). In this trial immunostaining for CD3, CD8, and CD45RO markers and high-resolution scanning of the stained slides and quantification of digital images (Definiens) is performed [132]. Genomic study of the tumor to assess the MSI status, the presence of a KRAS and BRAF mutations will be conducted for the same patients. The MISIPI study is a clinical trial focused on the prognostic and predictive value of the immunoscore including CD3, CD8, CD20, FoxP3 and CD163 and PD-L1 in patients with advanced melanoma treated with Ipilimumab with the follow-up of 2–3 years [133]. The immunoscore is evaluated in metastatic lymph node tissue which are constitutively rich in CD3+ and CD20+ lymphocytes, and correlated it with outcomes and response to the treatment. Lymph nodes often are the unique site of metastatic disease and are more accessible to biopsy than distant metastasis [134]. Immunoscore (CD3, CD8, in tumor center and margin) has been evaluated the resected metastasis of 116 brain metastatic patients, supporting the major prognostic value of Immunoscore in brain metastasis. Immunoscore showed significant correlation with survival prognosis (27 vs. 10 months; p < 0.001). The prognostic impact of Immunoscore was independent from established prognostic parameters at multivariable analysis (HR 0.612, p < 0.001) [135].

There are two ways to up-regulate PD-L1 expression in solid tumors: by constitutive, oncogenic signaling and by an adaptive, inducible mechanism, both of which may be operative in different tumor types. The concept of PD-L1-mediated adaptive immune resistance was developed when the observation was made that PD-L1 expression in melanoma is often focal, and geographic, and almost always immediately adjacent to TIL. When this finding was explored further, it was apparent that while TILs are necessary, they are not sufficient for PD-L1 expression in melanomas, leading to the hypothesis that it was functional differences in TILs that accounted for the differential PD-L1 expression by tumor cells [136]. When whole transcriptome analysis was performed comparing PD-L1+ vs. PD-L1(−) melanomas, functional groups of differentially expressed genes were observed, including an IFN-gamma/Th1 signature, a CD8 T-cell signature, and numerous checkpoint molecules [137]. Taken together, these findings indicate that PD-L1 expression when observed adjacent to TIL reflects an adaptive immune resistance to an ongoing anti-tumor immune reaction. This mechanism is not dependent on the BRAF mutational status of the melanoma [138].

PD-1/PD-L1 blocking antibodies work by thwarting this mechanism of adaptive immune resistance, and essentially protect the ongoing host immune response against tumor. Because the anti-PD-1/PD-L1 antibodies are thought to act locally within the tumor itself, PD-L1 expression in pre-treatment tumor specimens has been explored as a possible “biomarker” to help predict which patients will respond to anti-PD-1/PD-L1 therapies. In a series of 49 patients with melanoma and other solid tumors, all clinical responders had PD-L1-expression on the surface of their tumor cells in their pre-treatment biopsies [138]. Similar results have since been shown in many other trials in multiple tumor types using anti-PD-1 or anti-PD-L1. While it is now recognized that a proportion of PD-L1 negative patients may also respond to these therapies, the objective response rate in PD-L1(+) patients exceeds PD-L1(−) patients 3–4 fold [139]. In ongoing clinical trials, PD-L1 expression in the tumor microenvironment is also being explored as it relates to progression-free survival and overall survival.

When other single factors in the tumor microenvironment such as PD-L1 expression by immune cells, expression of PD-1, CD4:CD8 ratio, and the presence of TIL were explored as markers of response to anti-PD-1, PD-L1 expression by tumor cells remained the strongest single predictor of response to anti-PD-1 therapy [140]. Other investigators have identified CD8 density at the leading edge of the tumor as the single factor most closely associated with response. The potential predictive value of immune factors in pre-treatment tumor samples in patients receiving anti-PD-1 may be further enhanced by studying multiple measures associated with the adaptive immune resistance phenomenon, rather than a single factor [141]. Such markers are highly desirable as some patients will demonstrate a delayed response or even paradoxical tumor growth before regression, and if they can be identified as likely clinical responders, treatment can be continued with confidence. Further, there are potential immune-related side effects with checkpoint blockade agents, so biomarkers that can help shift the cost:benefit ratio for patients are highly sought after. In the future, additional characterization of the pre-treatment and on-treatment tumor microenvironment will likely facilitate the rational design of combinatorial treatments with anti-PD-1/L1 agents and help to further improve patient outcomes.

The role of BRAF and NRAS in the immunological landscape of melanoma has been poorly investigated and the effect of their mutations on global gene expression remains to be fully elucidated. Two immune phenotypes have been described in melanoma: a Th1 phenotype, characterized by the expression of melanocytic lineage specific transcripts, a better prognosis and higher responsiveness to immunotherapy, and a Th17 phenotype, associated to enhanced cellular motility, poorer prognosis and a more undifferentiated status. A classification that differentiate melanoma metastases based on genes consistently expressed in vivo and in vitro has been previously identified [142] and named transcriptional adjustments related to amplification/deletions (TARA), which resembles the pattern created in breast cancer with Luminal or Basal-like classification. One-hundred-thirteen metastatic melanoma tissues have been classified according to TARA; TARA class A displayed a classic Th17 phenotype, with expression of IL-17, IL-1, TNF-alpha and IL-23, while TARA class B identified a typical Th1 phenotype with expression of STAT1, GBP1 and CXCL10. Authors have also examined patterns of BRAF and NRAS mutations in these 113 metastatic tissues: 29 % were wild-type, 59 % had a BRAF mutation and 12 % a NRAS mutation. BRAF and NRAS mutations did not affect the transcriptome at global level, however, BRAF-specific genes discriminated Th1/good vs. Th17/poor phenotype. The association between BRAF mutation and poor phenotype was particularly strong in samples displaying low BRAF mRNA expression. Functional interpretation of genes differentially expressed in metastases displaying high and low BRAF mRNA revealed IL-2 and JAK/Stat among the top canonical pathways. This suggests that BRAF mRNA expression may also play a role in the association to an immune phenotype in melanoma.

Uveal melanoma is the most common intraocular malignancy in the adults, originating from the melanocytes located in the human uvea. The most frequent site of onset are the ciliary body and choroid (95 % of cases), more rarely the iris (5 %) is affected. Uveal melanoma patients have a disease-specific 5-year survival around 40 %. Driver mutations in *GNAQ* and *GNA11* can be found in about 80 % of uveal melanomas, which is thereby genetically different from cutaneous and conjunctival melanoma [143, 144]. While localized disease can be treated with surgery or radiotherapy, there is lack of effective treatments for metastatic uveal melanoma. A meta-analysis of 40 clinical trials (only 1 of them being a phase 3 randomized trial) enrolling more than 800 patients showed a less than 5 % ORR and a median PFS of 1.8–7.1 months [145]. Due to the critical role of the RAF/MEK/ERK signaling pathway in uveal melanoma, clinical trials with specific inhibitors such as sulemetinib, sorafenib and MEK162 are ongoing [146]. Uveal melanomas do express some tumor antigens such as GP100, MART-1, tyrosinase and TRP-1 [147]. These tumors also create an inflammatory microenvironment and have a functionally relevant PD-L1 expression, providing a rationale for immunotherapy. In expanded access programs and retrospective studies, ipilimumab showed some activity in patients with metastatic uveal melanoma. The largest dataset included 82 unselected patients, in which disease control rate was 30 % with a median PFS of 3.6 months and a 1-year-survival rate of 31 % [148]. Recently, a phase II trial by DeCOG (Dermatologic Oncology Cooperative Group) which aimed to prospectively evaluate the efficacy and safety of ipilimumab in patients with cutaneous melanoma and rare subgroups was published [149]. In this trial, 53 patients with uveal melanoma were included, with 85 % of cases having at least one prior systemic therapy. In these patients, however, ipilimumab did not show any relevant activity. No objective response has been reported, with median PFS was 2.83 months and only 22 % of patients alive at 1 year. The presence of brain metastases and of a prior treatment was not associated with decreased survival. Conversely, pretreatment LDH levels (low vs. high) and absolute lymphocyte count (high vs. low) were associated with improved survival. The reasons why ipilimumab showed such limited activity in metastatic uveal melanomas are not clear. To further evaluate immune-checkpoint blockade in uveal melanoma, a new murine model was established using stably transduced melan-a cells expressing an activating mutation of the uveal melanoma oncogene *GNAQ*. In this model, dual therapeutic immune checkpoint blockade (anti-CTLA-4 and anti-PD-1) is needed in order to delay tumor growth, showing no activity if either agent was administered as monotherapy (Schilling and Griewank unpublished). To further evaluate the role of immune-checkpoint blockade in uveal melanoma, additional preclinical studies might provide new insight into the interaction of uveal melanoma and the host’s immune system. However, only prospective, randomized trials will be able evaluate the full potential of immunotherapy in metastatic uveal melanoma.
